# Symptomatic Pituitary Metastasis as Initial Manifestation of Renal Cell Carcinoma: Case Report and Review of Literature

**DOI:** 10.1155/2020/8883864

**Published:** 2020-08-18

**Authors:** Gunjan Y. Gandhi, Russell Fung, Patrick E. Natter, Raafat Makary, K. C. Balaji

**Affiliations:** ^1^Division of Endocrinology, University of Florida College of Medicine-Jacksonville, Jacksonville, FL, USA; ^2^Department of Medicine, University of Florida College of Medicine-Jacksonville, Jacksonville, FL, USA; ^3^Department of Radiology, University of Florida College of Medicine-Jacksonville, Jacksonville, FL, USA; ^4^Department of Pathology and Laboratory Medicine, University of Florida College of Medicine-Jacksonville, Jacksonville, FL, USA; ^5^Department of Urology, University of Florida College of Medicine-Jacksonville, Jacksonville, FL, USA

## Abstract

Metastasis to the pituitary gland is extremely rare (∼2% of sellar masses). Clinical, biochemical, and radiologic characteristics of pituitary metastasis are poorly defined and can be difficult to diagnose before surgery. We present an unusual case with pituitary metastasis as the first manifestation of renal cell carcinoma (RCC). A 70-year-old male presented with acute onset of weakness, dizziness, diplopia, and progressively worsening headache. The initial CT head revealed a heterogeneous sellar mass measuring 2.8 × 1.9 × 1.7 cm. A follow-up MRI showed the sellar mass invading the right cavernous sinus. The presumptive diagnosis was a pituitary macroadenoma. Physical examination revealed bilateral 6th cranial nerve palsy and episodes of intermittent binocular horizontal diplopia. Hormonal testing noted possible secondary adrenal insufficiency (AM serum cortisol: 3.3 mcg/dL, ACTH: 8 pg/mL), secondary hypothyroidism (TSH: <0.01 mIU/L, FT4: 0.7 ng/dL), secondary hypogonadism (testosterone: 47 ng/dL, LH: 1.3 mIU/mL, and FSH: 2.3 mIU/mL), and elevated serum prolactin (prolactin: 56.8 ng/ml, normal: 4.0–15.2 ng/ml). IGF-1 level was normal at 110 ng/mL (47–192 ng/mL). The patient was discharged on levothyroxine and hydrocortisone therapy with plans for close surveillance. However, his condition worsened over the next three months, and he was subsequently readmitted with nausea, vomiting, and hypernatremia secondary to diabetes insipidus. Repeat MRI pituitary showed an interval increase in the size of the sellar mass with suprasellar extension and a new mass effect on the optic chiasm. The sellar mass was urgently resected via a trans-sphenoidal approach. The tumor was negative for neuroendocrine markers and pituitary hormone panel, ruling out the diagnosis of pituitary adenoma and triggered workup for metastatic renal cell carcinoma, clear cell type. The diagnosis of renal cell carcinoma was confirmed by the diffuse and strong staining for renal cell carcinoma markers (Pax-8, RCC-1, and CD10). A follow-up CT scan noted large right renal mass measuring 11 × 10 × 11 cm. The patient underwent a cytoreductive robotic right radical nephrectomy for WHO/ISUP histologic grade II clear cell RCC, stage pT2b pNX pM1. He subsequently received fractionated stereotactic radiotherapy to the pituitary gland. He is presently stable with no radiological evidence of progression or new intracranial disease on subsequent imaging. Pituitary metastasis most commonly occurs from breast, lung, or gastrointestinal tumors but also rarely from renal cell carcinoma. Biochemical findings such as panhypopituitarism, acute clinical signs such as headache, visual symptoms, and diabetes insipidus and interval increase in sellar mass in a short time interval should raise suspicion for sellar metastasis.

## 1. Introduction

The sellar region is an uncommon site of metastatic disease. In 1857, L. Benjamin first described a case of metastasis to the pituitary gland in an autopsy of a patient with melanoma [1]. Since then, clinicians have become more aware of considering the pituitary gland as a potential site for metastatic disease [2, 3]. However, pituitary metastases are uncommon, occurring in 1% to 4% of all cancer patients in large autopsy studies, the most frequent primary tumors being breast and lung cancer [2, 4–6]. Most often, they are part of a generalized metastatic spread, especially skeletal [1, 7–10]. They tend to affect patients in the sixth or seventh decade of life [11, 12], with no clear sex predominance. Infrequently pituitary metastasis has been noted to be the first manifestation of an occult primary tumor or the only site of metastasis [7, 9, 10] and occurs in younger adults [7, 13].

Metastasis to the pituitary is often asymptomatic. Diabetes insipidus has traditionally been thought to be the most common symptom of pituitary metastases [7, 14], particularly when the posterior pituitary gland is affected. Anterior pituitary insufficiency and cranial nerve deficits have been less frequently described at presentation [15–17]. This has been attributed to a predisposition of metastasis to the posterior lobe and the pituitary stalk. Mild elevation of prolactin at levels less than 200 ng/ml, consistent with stalk compression, is not uncommon in pituitary metastasis [18]. There are no *sine qua non* radiological and clinical manifestations of sellar metastasis [19–21]. They may often mimic a pituitary adenoma [1, 9, 15, 16] or a variety of sellar lesions such as granuloma, abscess, cyst, aneurysm, tumor, and apoplexy [9, 18, 22–26]. This is especially true if there is no existing evidence of malignancy in the patient. Best practices of pituitary metastases treatment are not available as there is a lack of standardized therapy guidelines [10, 27–30]. The primary malignancy determines long-term prognosis. Local therapy aiming at symptom relief may be beneficial, and ensuring optimizing quality of life in accordance with patient's values and preferences is critical.

Renal cell carcinoma is a rare cause of pituitary metastases, with only a few cases having been reported thus far [28]. Anterior pituitary dysfunction may be more prevalent, and diabetes insipidus less commonplace in patients with renal cell carcinoma metastatic to the pituitary compared to ones associated with other primary malignancies [19, 27, 28]. We describe a case of a patient presenting with symptomatic pituitary metastasis from renal cell carcinoma. It is particularly revealing that pituitary metastasis was the first manifestation of cancer, and the absence of a known history of malignancy confused the diagnosis. We further describe the incidence, pathogenesis, diagnosis, and management of patients with pituitary metastasis by following the methods of Zhao et al. [31], He et al. [2], Castle-Kirszbaum et al. [3], and Gopan et al. [32].

## 2. Case Presentation

**Figure 1:**
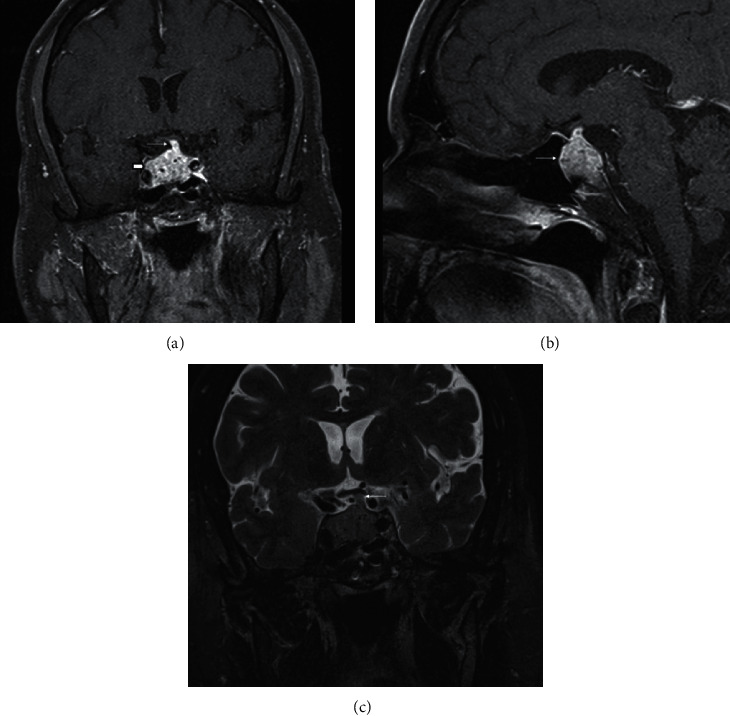
(a) Coronal postcontrast fat-saturated image demonstrates a heterogeneous sellar mass, which expands the sella (short thick arrow). There is also a slight suprasellar extension. The pituitary stalk is also involved and thickened (thin arrow). (b) Sagittal postcontrast fat-saturated image also demonstrates the heterogeneous sellar mass (thin arrow). (c) Coronal T2 image shows a mild mass effect on the optic chiasm from the thickened pituitary stalk (thin arrow).

A 70-year-old male presented with a 5-day history of double vision that was described as horizontal diplopia. It was most often provoked with a quick lateral gaze and resolved with rest. He noted frontal headache, which was dull aching and always present. He noted some relief of pain with NSAIDs but never to full resolution. He also noted decreased energy and a feeling of weakness in legs. Past medical history was notable for diabetes mellitus not requiring treatment with medications, gastroesophageal reflux disease, hyperlipidemia, and tachycardia. He previously had meniscus surgery on his knee. Physical exam revealed bilateral 6th cranial nerve palsy and episodes of intermittent binocular horizontal diplopia. Initial CT head revealed a sellar mass involving the right cavernous sinus measuring 2.8 × 1.9 × 1.7 cm, confirmed by MRI demonstrating a heterogeneous sellar mass invading the right cavernous sinus with a leftward deviation of the infundibulum. There was a small amount of suprasellar extension with mild mass effect on the optic chiasm (Figures 1(a)–1(c)). The presumptive diagnosis was a pituitary macroadenoma. Hormonal testing noted possible secondary adrenal insufficiency (AM serum cortisol: 3.3 mcg/dL, ACTH: 8 pg/mL), secondary hypothyroidism (TSH: <0.01 mIU/L, FT4: 0.7 ng/dL), secondary hypogonadism (testosterone: 47 ng/dL, LH: 1.3 mIU/mL, and FSH: 2.3 mIU/mL), and hyperprolactinemia with mildly elevated prolactin levels (prolactin: 56.8 ng/ml, normal: 4.0–15.2 ng/ml). IGF-1 level was normal at 110 ng/mL (47–192 ng/mL). No acute neurosurgical involvement was planned. Formal visual field testing did not show visual field defects consistent with pituitary mass. The patient was discharged on hormone replacement therapy with levothyroxine 100 mcg daily and hydrocortisone 20 mg in the morning and 10 mg in the afternoon with plans for consideration of testosterone replacement therapy as an outpatient. His vision improved to some degree during hospitalization.

**Figure 2:**
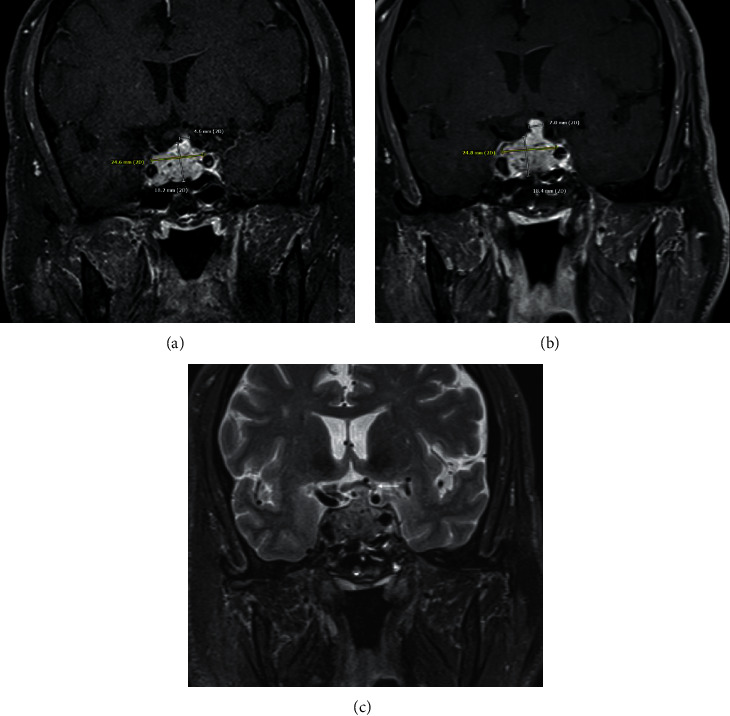
Coronal postcontrast image demonstrates the size of the mass upon initial imaging (a) also including the measurement of the mildly thickened pituitary stalk. At the time of follow-up imaging (b), the mass has slightly increased in size, which is most notable at the pituitary stalk, which now measures 7 mm in thickness, previously 4.6 mm. Also, note the mild increased mass effect on the optic chiasm (thin arrow) at the time of follow-up imaging on the coronal T2 image (c).

Repeat MRI in 3 months noted interval increase in the bulk of the tumor extending along the infundibulum, now measuring 0.7 cm in transverse width compared to 0.4 cm previously, and there was resulting interval mild increased mass effect on the optic chiasm at its central and left aspect (Figures 2(a)–2(c)). Besides, there was an interval increase in the tumor in the sella. The mass continued to extend to the right lateral aspect concerning for right cavernous sinus invasion with erosion into the clivus inferiorly. As a result of the growth of the sellar mass, neurosurgery planned surgical intervention. A few days later, the patient presented to the emergency room with severe acute headache, nausea, vomiting, polyuria, and polydipsia. The examination noted nystagmus and decreased abduction of the right eye. Sodium was mildly high, confirming diabetes insipidus requiring short-term DDAVP treatment. A repeat MRI did not indicate a significant change of the pituitary mass compared to MRI done a few days earlier. He was started on stress dose steroids in the form of hydrocortisone 50 mg IV every 8 hours. He underwent emergent endoscopic endonasal trans-sphenoidal resection of the pituitary lesion. The tumor was firm and hemorrhagic. Preliminary intraoperative pathology (frozen section) was suggestive of pituitary adenoma and tumor debulking performed as much as safely possible. He continued to have transient polyuria following surgery, requiring DDAVP until his discharge from the hospital. DDAVP did not have to be maintained as an outpatient as diabetes insipidus was mild.

**Figure 3:**
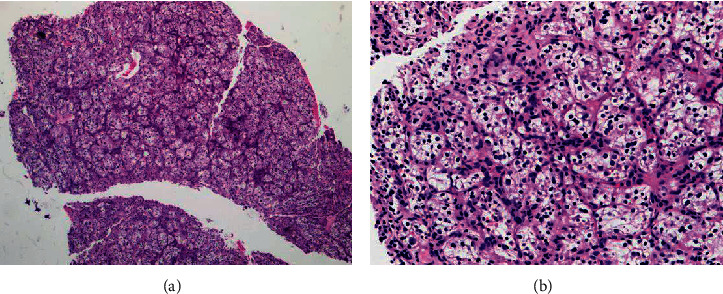
Pituitary tumor excision showing tumor nests of clear cells separated by fibrovascular septa (H&E stain: (a) ×2.5; (b) ×10).

**Figure 4:**
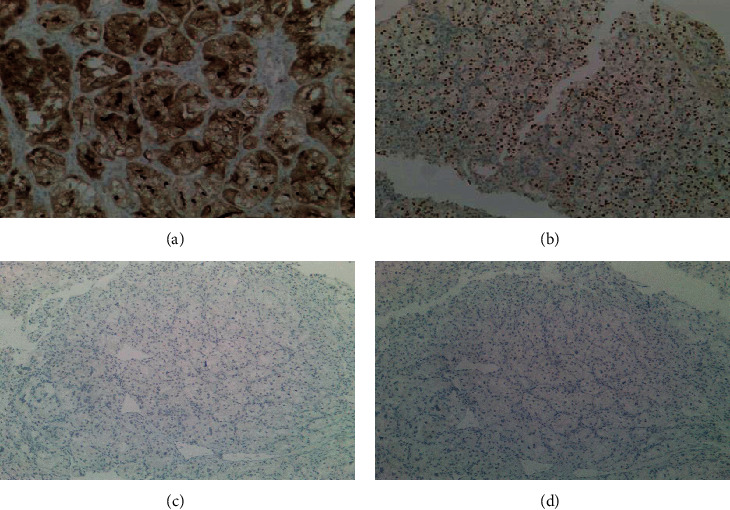
Immunohistochemical stains positive for renal cell carcinoma (a) and (b) and negative for neuroendocrine markers (c) and (d). (a) CD10 immunostain (×10). (b) PAX-8 immunostain (×5). (c) Synaptophysin (×2.5). (d) Chromgranin (×2.5).

Surprisingly, pathology of the resected pituitary mass after proper fixation showed metastatic renal cell carcinoma (Figures 3 and 4). The tumor histology was characterized by a nested Zellballen pattern of cells (on hematoxylin&eosin and reticulin stains), separated by a delicate network of thin wall capillary vasculature. The tumor cells had relatively defined cell borders, clear and eosinophilic cytoplasm, round slightly pleomorphic nuclei with small prominent central nucleoli in several of the tumor cells. Mitoses were inconspicuous, and no tumor necrosis was seen. The tumor cell histomorphology was unusual for pituitary adenoma and triggered the immunohistochemical characterization of the tumor lineage (phenotype). Immunohistochemical stains were negative for neuroendocrine markers (chromogranin, synaptophysin, and neuron-specific enolase), pituitary hormones panel (TSH, ACTH, GH, prolactin, and nonspecific staining for FSH), and S100 and focally positive for CD56. The immunostain results were inconsistent with the diagnosis of pituitary adenoma. The clear cell morphology suggested metastatic renal cell carcinoma of clear cell type, which was confirmed by the diffuse and strong positive immunostains for renal cell carcinoma markers (Pax-8, RCC-1, and CD10). The Ki-67 proliferation marker labeled approximately 10% of the tumor cell nuclei.

**Figure 5:**
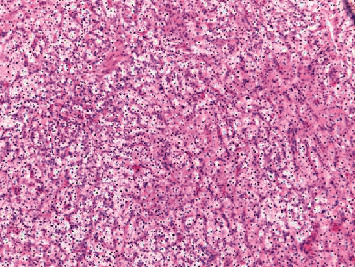
Right kidney, radical nephrectomy: clear cell renal cell carcinoma, WHO/ISUP histologic grade 2. The tumor measures 10.5 cm in greatest dimension, limited to kidney.

Due to pathology findings, a CT scan of the abdomen was done that noted large heterogeneously enhancing solid mass, arising from the middle and lower thirds of the right kidney with prominent stellate central necrosis, measuring approximately 11 × 10 × 11 cm. There was significant hyperemia in the perinephric fat with prominent vessels tracking along the lateral conal fascia. Haziness in the perinephric fat slightly concerned for early tumor spread. A 2 cm enhancing nodule in the left adrenal gland was also seen. PET/CT scan showed the renal mass to be not FDG avid. Minimal FDG uptake was seen of the left adrenal mass. As a result, a cytoreductive robotic right radical nephrectomy was performed, which confirmed WHO/ISUP histologic grade II clear cell renal cell carcinoma on pathology, stage pT2b pNX pM1 (Figure 5). The carcinoma did not involve margins and there was no lymphovascular invasion.

**Figure 6:**
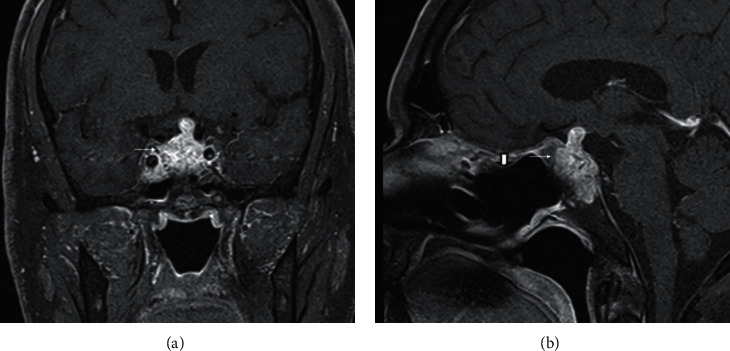
Most recent postoperative scan demonstrates residual enhancing heterogeneous mass in the sella (thin arrow) on the coronal postcontrast image (a). The sagittal postcontrast image (b) also demonstrates the residual heterogeneous mass (thin arrow). Also, note the postsurgical changes in the adjacent sphenoid sinus and posterior ethmoid air cells from a trans-sphenoidal approach to the sella (short thick arrow).

Repeat MRI of the sella noted subtotal resection of the pituitary mass. Repeat visual field testing indicated no defects. The patient subsequently received fractionated stereotactic radiotherapy to the pituitary metastasis at a dose of 30 Gy in 5 fractions. Repeat MRI 3 months after surgery and one year after surgery noted the sellar mass was stable with the thickened infundibulum causing mass effect upon the optic chiasm (Figures 6(a) and 6(b)). He has continued to require levothyroxine along with hydrocortisone and has been subsequently started on intramuscular testosterone injections. Subsequent imaging of the renal lesion has noted right retrocaval enhancing lesion concerning for metastatic lymph node from patient's previously identified right renal cell carcinoma along with slight interval enlargement of an enhancing left adrenal gland nodule with the possibility of a metastatic adrenal lesion. He has been offered options of tyrosine kinase inhibitor, immunotherapy with or without tyrosine kinase inhibitor, or surveillance. Stereotactic radiation to the adrenal area has also been discussed. The role of local treatment for the oligometastatic disease has been reviewed with the patient. The patient prefers to maintain his current quality of life and avoid additional treatment with potential side effects for now. He has elected surveillance for now and is clinically doing well.

## 3. Discussion

Our case illustrates the importance of considering a broad differential diagnosis of a sellar mass. The differential diagnosis should include consideration of more commonly known conditions such as pituitary adenoma but also less frequent entities such as cysts, abscesses, granulomas, apoplexy, trauma, meningiomas, craniopharyngiomas, and aneurysm as well as pituitary metastasis amongst others. It is essential to consider that sellar symptoms may be the first manifestation of neoplastic diseases, such as in our patient in whom renal cell carcinoma was diagnosed after surgical pathology noted pituitary metastasis from renal cell carcinoma. It can closely mimic a benign pituitary adenoma symptomatically, as demonstrated in our case. Any clinical, biochemical, or imaging findings of pituitary pathology in a patient with known cancer, especially lung and breast, should raise suspicion for metastasis to the sella. Moreover, the development of diabetes insipidus or ophthalmoplegia from any pituitary lesion is suggestive of metastatic disease even in patients with no known primary malignancy. As in our case, an interval increase in sellar mass in a short time interval should also raise suspicion for sellar metastasis. Ultimately, pathology remains the gold standard for the diagnosis of pituitary metastasis.

Pituitary metastasis is rare. The incidence of pituitary metastasis among all intracranial metastases is 0.87%; it is 1.9% among all autopsied cancer cases in a large autopsy study [2]. In females, the most likely source of pituitary metastasis is the breast, while in men, metastases tend to be from the lung [33]. Two-thirds of metastasis to the sella originate from these primary sites, which could be as these are two of the more common malignancies. Although it is difficult to know the exact reason for affinity towards the pituitary for spread, the pituitary gland is rich in prolactin receptors that may attract cancerous breast cells [6]. Also, the gastrointestinal tract, prostate, kidney, thyroid, and pancreas primary tumors are possible sources of pituitary metastases [2, 3]. Renal cell carcinomas tend to very uncommonly spread to the pituitary gland [28, 32, 34, 35]. In a case series, Gopan et al. noted the average age of presentation for renal cell carcinoma metastatic to the pituitary gland to be 61 years, with most being male [32]. Of those with known renal cell carcinoma, the average interval between diagnosis and pituitary metastasis was approximately eight years [32]. Pituitary metastasis may be the initial presentation of an unknown primary tumor in up to 20–30% patients [36].

The mechanism of metastasis to the pituitary gland is unclear. Still, it likely involves the following: (1) migration to the pituitary gland through a portal vessel system, (2) migration to the pituitary gland or sellar region via the internal carotid, (3) migration via the suprasellar cistern into the pituitary gland, and (4) migration from the skull base to the pituitary gland [5, 10, 29]. The most commonly involved site is the posterior lobe of the hypophysis (69–79%), followed by the anterior pituitary lobe, and then both the anterior and posterior hypophysis, and finally, the pituitary stalk [37].

Most patients with pituitary metastasis are asymptomatic [5, 8, 15, 38]. Some presenting symptoms include diabetes insipidus, hypopituitarism, visual field defects, ophthalmoplegia, and headache. Renal cell carcinoma has been noted to be strongly associated with visual field defects likely due to extension to the suprasellar cistern resulting in the compression of the optic chiasm and optic nerve [32]. Brisk worsening of vision may be a more useful gauge to separate sellar metastasis from pituitary macroadenomas as both conditions seem to have a high incidence of visual field defects [2, 39, 40]. Encroachment of cranial nerves in the cavernous sinus (three, six, four, and five) should raise suspicion for pituitary metastasis. Case series of patients with pituitary metastasis have noted cranial neuropathies to occur more commonly than in patients with pituitary macroadenomas [2, 3, 41]. Seizures associated with pituitary metastasis from small cell lung cancer have been described [42]. Headache is relatively common, seen in about one out of five patients with sellar metastasis [2]. No defining characteristics of headache have been noted in patients with pituitary metastasis. As renal cell carcinoma can have increased blood flow, there have been reports of pituitary apoplexy [43] and intraventricular hemorrhage causing hydrocephalus [44].

Diabetes insipidus is the most common symptom of pituitary metastases, particularly when the posterior lobe of the gland is affected. A large case series of pituitary metastases demonstrated that the posterior lobe was involved in more than 80% [8]. The main reason for this frequent involvement of the posterior pituitary lobe is related to its extensive contact with the adjacent dura mater and its direct arterial supply from the hypophyseal arteries, contrasting with the anterior lobe, which is supplied from the hypophyseal portal system [20]. Since diabetes insipidus is an infrequent symptom in pituitary adenoma, it can be particularly helpful in differentiating a metastatic tumor from a pituitary adenoma [9]. When compared to other pituitary metastasis, renal cell carcinoma tends to present more commonly with hypopituitarism and less often with DI [32, 45].

In a pooled study of patients with metastasis to the pituitary gland, hypopituitarism was seen in about a quarter of the patients [2]; this may be due to mass effect on the pituitary infundibulum and anterior pituitary lobe. An increase in prolactin is seen in a vast majority of patients with pituitary metastasis [46], but the degree of elevation is mild. It may be likely be related to the “stalk effect” rather than an actual prolactin-secreting tumor. Also, the prevalence of elevated prolactin level is relatively high in pituitary macroadenomas [46] and thus not helpful to differentiate between the two. When compared to nonfunctioning macroadenomas, deficiencies of anterior pituitary hormones, including ACTH, TSH, FSH, and LH, were significantly more common among patients with metastatic disease [46]. Isolated cases of neuroendocrine tumors (lung and pancreas) secreting GHRH and ACTH have been reported with functioning pituitary metastasis, causing acromegaly and Cushing's syndrome [47–49].

Pituitary metastasis appearance on imaging tends to overlap with other tumors, especially pituitary macroadenoma, and the imaging findings of metastasis are often nonspecific [50–53]. Invasion of adjacent structures such as the bony sella and cavernous sinuses is nonspecific and can be seen with pituitary metastasis or pituitary macroadenoma [50]. Multiple appearances have been reported previously. Metastasis can present with a “dumbbell” shaped mass with sellar and suprasellar components with an indentation at the level of the sellar diaphragm [17, 54]. It has been described that the indentation may be more prominent with metastasis at the sellar diaphragm due to rapid growth as compared to a pituitary macroadenoma, which could slowly expand the sellar diaphragm due to slower growth [17, 54]. Pituitary metastasis can also primarily involve the pituitary stalk with associated mass or thickening involving the stalk. One series demonstrated a pituitary stalk lesion in approximately 63.3% of patients. Metastatic disease in one radiological case review series was the most common adult infundibular tumor [55]. Another pattern of involvement that has been described is the pituitary infundibulum as a visible linear enhancement at the posterior inferior border of metastasis with a suprasellar component [17]. Diabetes insipidus, when associated with sellar metastasis, may be visible as a thickened pituitary stalk along with the absence of the usual high T1 signal intensity in the posterior pituitary lobe [54].

Treatment of metastasis to the pituitary requires a multidisciplinary approach. Treatment generally includes surgery, postoperative stereotactic radiosurgery, chemotherapy, and hormone replacement therapy. If a patient has an initial clinical presentation of deteriorated visual acuity and visual field impairment, which indicates compression of the optic nerve, surgery is often the first choice. The endoscopic endonasal trans-sphenoidal approach is the preferred surgical approach and can help improve headache, visual field defects, and cranial nerve deficits [29, 56]. The goal of surgery is to decrease the compression of optic chiasm caused by the often firm and vascular tumor and symptom relief as well as improving quality of life rather than improving survival [5, 10, 29, 57]. Surgery is often technically difficult due to possible invasion of tumor into cavernous sinuses. The gamma knife is a stereo-directional radiotherapy method that is used when lesions do not compress or only slightly compress optic nerves and optic chiasm. Piedra et al. [58] noted that the gamma knife approach could improve the symptoms of diabetes insipidus and minimize the deterioration of visual acuity and visual fields without causing hypopituitarism. Stereotactic radiosurgery thus represents an effective, safe, and noninvasive means of ameliorating the symptoms of pituitary metastasis in patients with limited survival. Hormone replacement therapy is critical under the guidance of an endocrinologist. Radiotherapy and chemotherapy are appropriate for patients with multiple metastatic lesions. Due to a better understanding of biology of renal cell carcinoma, clinicians now have a larger arsenal of targeted therapeutic options and immunotherapies available to them. One novel option is a tyrosine kinase inhibitor such as cabozantinib. Other options include a combination of nivolumab (antiprogrammed cell death 1 (PD-1)) and ipilimumab (anticytotoxic T-lymphocyte-associated antigen 4 (CTLA-4)). Combined use of atezolizumab (anti-PD-ligand 1 (PD-L1)) and bevacizumab (antivascular endothelial growth factor (VEGF)) has also been an effective therapy [59]. A recent network meta-analysis and systematic review by our group on first-line therapy for metastatic RCC demonstrated that a combination of pembrolizumab plus axitinib might be the preferred option based on efficacy and side effect profile compared with avelumab plus axitinib or atezolizumab plus bevacizumab [60]. Yang et al. [61] reported a case of pituitary metastasis from renal cell carcinoma; although the pituitary metastasis progressed, extracerebral metastasis showed a partial response to sorafenib treatment. Therefore, chemotherapy alone does not improve the treatment course of pituitary metastasis; surgery and postoperative radiotherapy are required. Some chemotherapeutic and immunotherapeutic regimens cross the blood-brain barrier and may provide a survival advantage, although evidence is limited to case reports [62, 63]. In addition to the role of cytoreductive nephrectomy in patients with metastatic renal cell carcinoma, there may be a role for treating primary renal carcinoma in presence of oligometastatic or progressive metastatic disease. There is a case report of improved outcome following partial nephrectomy in a patient with thyroid metastasis from renal cancer [64]. In addition to treating primary tumor in presence of metastatic renal cell cancer, the other strategy to improve outcome is metastasis-directed therapy especially in the setting of oligometastatic disease. Both surgical and radiotherapy approaches with or without adjuvant therapy such as immune check point inhibitors are described in the literature. However, currently there are no randomized phase III studies validating the metastases-directed local or regional therapy in patients with metastatic renal cell cancer [65–68]. While both minimally invasive and open approaches are effective in performing nephrectomy, the minimally invasive approach offers advantages of fewer complications and quicker postoperative recovery [69]. Moreover, available surgical expertise influences the choice of method and the surgeon at our institution was the first to perform and report on robotic nephrectomy [70].

The prognosis of pituitary metastasis, in general, is poor. Fassett et al. [52] retrospectively reviewed 36 cases of pituitary metastasis and found the average survival to be six months. Another study by Lin et al. noted a median survival of 4 months after diagnosis. Solitary or small metastasis, functional performance status, younger age, local therapy, including radiation therapy, and a longer interval from diagnosis of malignancy to pituitary metastasis have been linked to prolonged survival [57, 71]. With regards to specific primary malignancies metastatic to the pituitary, patients with renal cell carcinoma and melanoma survived longer when compared to patients with lung, colon, breast, prostate, liver, nasopharyngeal, and stomach malignancies [72].

In conclusion, sellar metastasis is uncommon and challenging to diagnose. Biochemical findings, such as panhypopituitarism, acute clinical signs such as headache, visual symptoms, and diabetes insipidus, and particularly rapid interval increase in pituitary mass in a short period, should raise suspicion for sellar metastasis. As in our case, pituitary symptoms may rarely be the first presentation of malignancy. Furthermore, in a patient with a known cancer history, there should be a heightened suspicion for metastasis to the pituitary gland. Treatment of pituitary metastases should be individualized and tailored to the patient's clinical condition, tumor histology, and presence of concomitant extrapituitary metastases. An important goal is symptom palliation and improving quality of life. A multidisciplinary approach to the disease (including involvement of an experienced radiologist, neurosurgeon, pathologist, endocrinologist, and urological oncologist) is necessary.
